# Loss of Microstructural Integrity in the Limbic-Subcortical Networks for Acute Symptomatic Traumatic Brain Injury

**DOI:** 10.1155/2014/548392

**Published:** 2014-02-20

**Authors:** Yanan Zhu, Zhengjun Li, Lijun Bai, Yin Tao, Chuanzhu Sun, Min Li, Longmei Zheng, Bao Zhu, Jun Yao, Heping Zhou, Ming Zhang

**Affiliations:** ^1^Department of Medical Imaging, First Affiliated Hospital of Medical College of Xi'an Jiaotong University, Xi'an 710061, China; ^2^Medical Imaging Centre, An Kang Central Hospital, 85-Jinzhou South Road, AnKang 725000, China; ^3^The Key Laboratory of Biomedical Information Engineering, Ministry of Education, Department of Biomedical Engineering, School of Life Science and Technology, Xi'an Jiaotong University, Xi'an 710049, China

## Abstract

Previous studies reported discrepant white matter diffusivity in mild traumatic brain injury (mTBI) on the base of Glasgow Coma Scale, which are unreliable for some TBI severity indicators and the frequency of missing documentation in the medical record. In the present study, we adopted the Mayo classification system for TBI severity. In this system, the mTBI is also divided into two groups as “probable and symptomatic” TBI. We aimed to investigate altered microstructural integrity in symptomatic acute TBI (<1 week) by using tract-based spatial statics (TBSS) approach. A total of 12 patients and 13 healthy volunteers were involved and underwent MRI scans including conventional scan, and SWI and DTI. All the patients had no visible lesions by using conventional and SWI neuroimaging techniques, while showing widespread declines in the fractional anisotropy (FA) of gray matter and white matter throughout the TBSS skeleton, particularly in the limbic-subcortical structures. By contrast, symptomatic TBI patients showed no significant enhanced changes in FA compared to the healthy controls. A better understanding of the acute changes occurring following symptomatic TBI may increase our understanding of neuroplasticity and continuing degenerative change, which, in turn, may facilitate advances in management and intervention.

## 1. Introduction

Mild traumatic brain injury (mTBI) is one of the most common injuries seen in emergency departments [1]. Approximately 15 to 30% of mTBI patients will experience kinds of cognitive and clinical symptoms known as the postconcussion syndrome (PCS) and do not resolve following the first 3 months after injury [2]. Furthermore, in some cases, the PCS-related complaints last several months to years, leading to even long-term disability [3]. TBI is one of the most consistent candidates for initiating the molecular cascades that result in Alzheimer's disease (AD), Parkinson's disease (PD), and amyotrophic lateral sclerosis [4]. The debate about the pathophysiology of mild TBI and its neurobehavioural symptomatology comes from psychogenic or physiogenic origin that has been strongly argued. Since these symptoms may be derived from more subtle neurological alterations and cannot be detected by only using conventional neuroimaging techniques such as conventional CT and MRI [5], recent upsurge of interest has been directed toward developing both diagnostic and prognostic biomarkers that can predict which individuals are relatively more likely to progress clinically.

Diffusion tensor imaging (DTI) is a technique that makes it possible to investigate white matter in vivo, since it provides information about white matter anatomy unavailable by any other method—either in vivo or in vitro [6]. Recent study suggests that subtle white matter abnormalities can be better detected by DTI than by conventional imaging [7]. These subtle abnormalities were potentially responsible for persistent postconcussive symptoms.

Taking the advantages of DTI, growing studies have focused on the correlation between structural integrity and mTBI recently. However, it was still unclear how these structural pictures evolved in mTBI patients. Arfanakis and coworkers firstly used DTI to investigate diffuse axonal injuries in acute mTBI (within 24 h of injury) and pointed out no significant mean diffusivity (MD) differences between mTBI patients and controls but attenuated fractional anisotropy (FA) in corpus callosum and the internal capsule in patients with mTBI [8]. Other study aimed to evaluate the correlation of the changes in FA and individual behavior performance. Niogi et al. found that subacute and chronic mTBI patients showed significant losses of FA in the left anterior corona radiate and uncinate fasciculus, which were significantly correlated with individual performances in attention control as well as memory, respectively [9]. And they inferred that FA can be used as a biomarker for neurocognitive function and dysfunction [10]. In addition, a longitudinal investigation demonstrated that mTBI was noted as a significant increase in fractional anisotropy and decrease in radial diffusivity in several left hemisphere tracts, and these trends even occurred after 3 to 5 months after injury [11]. Another study further explored that acute and chronic mTBI patients showed heterogeneous changes in the FA [12], and the main changes in FA for acute mTBI were significantly correlated with their postconcussion symptoms [13].

These discrepant findings do not necessarily conflict with each other, as there are many sources of variability inherent in MRI investigations that may contribute to the reported differences. Of various factors, one major effect is derived from the subtle difference in physiological state of mTBI. Previous study often adopted single indicators such as the Glasgow Coma Scale to classify different stages of TBI. However, this measure is often unreliable for some TBI severity indicators and the frequency of missing documentation in the medical record. In the present study, we adopted the Mayo classification system for TBI severity [14]. In this system, the mTBI is also divided into two groups as “probable and symptomatic” TBI.

In the present study, we aimed to investigate altered microstructural integrity in symptomatic acute TBI by using tract-based spatial statics (TBSS) approach. We hypothesized that (1) significant altered microstructural integrity occurred in the symptomatic acute TBI. (2) Though previous DTI studies have generally demonstrated lower integrity of white matter tracts in frontal and temporal regions in mTBI, we predicted the loss of integrity of limbic-subcortical in acute symptomatic TBI patients which were associated with emotional as well as executive dysfunction.

## 2. Materials and Methods

### 2.1. Participants

A total of 12 patients with acute symptomatic TBI (10 male, mean age 35.7, range 19–50) were recruited from the Emergency Department of An kang Central Hospital. Inclusion criteria were (1) first-episode, (2) symptomatic TBI defined according to the Mayo classification system for TBI severity, and (3) acute stage of TBI (<1 week). Exclusion criteria of symptomatic TBI were defined as current or previous drug or alcohol abuse, previous TBI, contraindications to MRI, penetrating injury, administration of sedatives/psychotropic/antiepileptic medication, spinal cord injury, neurological signs or multiple disabilities, history of psychiatric or psychological or neurological disease, MRI artifacts, and/or poor image quality. The control group comprised 13 healthy volunteers matched by the age, sex, and educational level. None had a history of neurological or psychiatric diseases. Their demographic characteristics were provided in Table 1. The study was approved by the local medical research ethics committee and institutional review board of local research ethics committees. All participants gave written informed consents.

### 2.2. Image Acquisition

The MRI protocol consisted of structural and functional images acquired on a 1.5 T Siemens Magnetom Avanto MRI scanner. Image acquisition was as follows: high-resolution T1-weighted anatomic images were obtained (TR = 1900 ms, TI = 1000 ms, TE = 2.8 ms, flip angle = 8°, 144 contiguous slices of 1.0 mm thickness, FOV = 256 × 256 mm^2^, and matrix = 256 × 256). T2-weighted images were obtained (TR = 4000 ms, TE = 79 ms, thickness = 5.5 mm, flip angle = 150°, FOV = 230 × 230 mm^2^, and matrix = 231 × 384). SWI images were obtained (TR = 49 ms, TE = 40 ms, flip angle = 15°, 72 contiguous slices of 2.0 mm thickness, FOV = 230 × 230 mm^2^, and matrix = 221 × 320). DTI scans were obtained (TR = 7300 ms, TE = 99 ms, thickness = 3 mm, directions = 30, FOV = 256 × 256 mm^2^, matrix = 128 × 128, Averages = 2, and *b*-value = 1,000/0 s/mm^2^).

### 2.3. Clinical Imaging

Patients were assessed by using standard T2 MRI to assess evidence of focal brain injury and SWI imaging to identify microbleeds, a marker of diffuse axonal injury. A senior consultant neuroradiologist reviewed all study MRI scans.

### 2.4. DTI Statistical Analyses

Diffusion data were preprocessed and analyzed using tools from the Oxford University Centre for Functional MRI of the Brain (FMRIB) software library (FSL Version 4.1). First, the b0 image of each subject was skull-stripped using the brain extraction tool. The data was corrected for subject motion and eddy-current-induced geometrical distortions, and the diffusion sensitizing gradients were rotated to correct for motion. Using FDT, the diffusion tensor model was fit to the data, from which FA images were calculated.

For tract-based spatial statistics (TBSS), all subjects' FA data was registered to a common space (the FA158 MNI space template) using a combination of affine and nonlinear registration. A mean FA image was created and eroded to a skeleton and threshold at FA > 0.2. Each subject's aligned FA data were then projected onto this skeleton and the resulting alignment-invariant representation of the central trajectory of white matter pathways was used for voxelwise statistical analysis (Randomize, 5000 permutations). The contrast TBI < controls was examined using threshold-free cluster enhancement (TFCE), with correction for multiple comparisons at *P* < 0.05.

## 3. Results

### 3.1. Demographic Results

Participants had to be between the ages of 18 and 60. Injuries were secondary to road traffic accidents (42%), assaults (42%), and falls (16%). Average scanning time after TBI was 5.5 days (range 1–7 days; SD 2.32). For a detailed list of means and demographic and injury characteristics, please see Table 1.

### 3.2. Clinical Imaging of Data

All of 12 patients with symptomatic TBI had a CT scan at the time of their emergency room visit, but none of the CT scans were deemed to contain trauma-related pathology by a nonblinded neuroradiologist. In addition, T2-weighted and SWI MRI images were reviewed by a neuroradiologist blinded to patient diagnosis. None of the patients had the well-defined evidence of lesion. Therefore, all the patients had no visible lesions by using conventional neuroimaging techniques.

### 3.3. Diffusion Tensor Imaging Scalar Analyses

**Figure 1:**
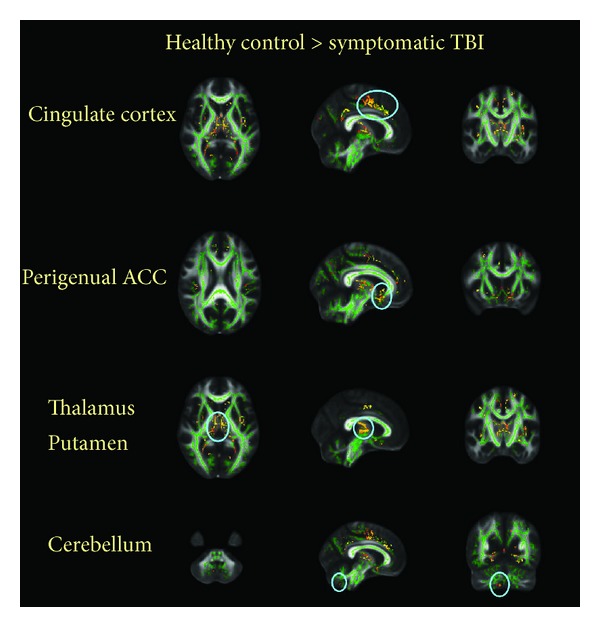
Group differences in mean FA. Symptomatic TBI patients showed a widespread decline in fractional anisotropy (FA) of gray and white matter throughout the TBSS skeleton (threshold at FA > 0.2, *P* < 0.05, corrected) overlaid on the TBSS skeleton (green).

Symptomatic TBI patients showed a widespread decline in fractional anisotropy (FA) of gray matter throughout the TBSS skeleton (shown in Figure 1). These regions included the bilateral frontal cortex (dorsal lateral prefrontal cortex, DLPFC; orbitofrontal cortex, OFC), the limbic system (bilateral subgenual and perigenual anterior cingulated cortex, sACC and pACC; bilateral posterior cingulate cortex, PCC; bilateral amygdala and parahippocampal gyrus), subcortical regions (bilateral caudate, claustrum, putamen, insula, and thalamus), occipital lobe (BA 7, 18, and 19), and temporal lobe (BA 20 and 37). In addition, the cerebellum also presented attenuated FA changes, primarily in the cerebellar lingual, declive, and uvula; please see Table 2. However, symptomatic TBI patients showed no significant enhanced changes in FA compared to the healthy controls.

The white matter of symptomatic TBI patients also exhibited FA decreases. Primarily, these regions located in the corpus callosum, limbic system (anterior cingulate, parahippocampal gyrus, and posterior cingulate) and sublobar areas (insula and extranuclear) as well as the frontal, temporal, occipital, and parietal lobe (precuneus) (see Table 3). However, symptomatic TBI patients showed no significant enhanced changes in FA compared to the healthy controls.

## 4. Discussion

In the current study, we observed a loss of structural integrity in multiple brain domains in acute symptomatic TBI patients (based on the Mayo classification system for TBI severity), who presented decreased fractional anisotropy values in widespread regions specially located in frontal lobe, limbic system, and sublobar areas compared with healthy controls.

In previous studies, DTI proved a sensitive technique that gave access in vivo to the structural integrity in mild TBI (mTBI) patients [7]. However, most of these studies generally induced discrepant findings only defining the mild TBI sample on the base of scores derived from Glasgow Coma Score (GCS). Single indicator such as the GCS was hardly to classify different stages of TBI. It is noted that about 6%–10% mTBI patients had visible lesions on CT [15]; these patients with positive CT scans tend to experience more neurobehavioral symptoms and poorer prognoses [16]. Therefore, this measures was often unreliable for some TBI severity indicators and the frequency of missing documentation in the medical record, leading to discrepant findings.

In the current study, we adopted the first-episode symptomatic TBI patients according to the Mayo classification system, based on available indicators including death due to TBI, trauma-related neuroimaging abnormalities, GCS, Posttraumatic Amnesia (PTA), loss of consciousness, and specified postconcussive symptoms. We supposed that, even in the stage of symptomatic TBI, there might be significant damage related to TBI that needed acute and proper treatment which was probably beneficial to the long-term prognosis.

Many studies reported discrepant white matter diffusivity in mTBI, increased, reduced, or unchanged. One of major factors was the unequal scanning time after mTBI. Recently, traumatic axonal injury (TAI) has been suggested encompassing not only the primary axonal damage specifically caused by shear/strain injury but also secondary alterations of white matter such as metabolic, hypoxic, and microvascular damage or excitotoxicity [17]. Moreover, axonal pathology is more pronounced in the acute phase of injury [18]. In the current study, we aimed to investigate the symptomatic TBI during the acute period after postinjury period that helped us to draw original patterns of TBI damage, which we can use as the baseline of longitudinal study to observe the recovery and/or deterioration of traumatic axonal injury.

Standard DTI analysis consisted of the placement of regions of interest, the tract-based spatial statistics. The former is restricted to assessment of the a priori defined regions, and only a small amount of the total white matter is usually investigated [9]. The latter allows the voxelwise assessment of all parts of large white matter tracts in an automated way [19], providing whole-brain voxelwise measures. It was clear that traumatic brain injury produced a complex pattern of diffuse axonal injury at wide range of brain regions and so it was hard to define regions with a priori information.

Fractional anisotropy (FA) is the DTI metric, which was commonly applied and measures preferential water diffusion along white matter tracts, and served as a usable marker of tissue integrity. In recent studies, FA has also been proposed as the most feasible biomarker of TAI and one of the best indicators of TBI severity [20, 21]. In the current study, we evaluated the structural integrity by observing the changes of FA values, which showed reductions in the TBI cohort. This finding was coincident with animal models of TBI, which have consistently indicated reduced anisotropic white matter water diffusion in the acute and semiacute injury stages [22]. But this finding appeared to be inconsistent with Ling et al. 's study, which proved increased FA in semiacute mTBI [23]. The possible reason was that the symptomatic TBI in the current study were different from their mild TBI, that all experienced an alteration in mental status, and the majority of the sample also experienced a loss of consciousness. The other reason was the earlier neuroimaging in the current study. If both reasons were all reasonable, we may suggest that the decreased FA probably indicated the original damage to the axon and the increased one indicated the recovery; we also suggested that an increase in FA may indicate more severe TBI correlated with poor clinical outcomes.

In the current study, we conducted the group comparison through FA skeleton maps, revealing significant FA reductions in the acute symptomatic TBI patients as compared to controls in the following areas: frontal lobe (DLPFC; OFC), the limbic-system (bilateral sACC and pACC, bilateral PCC, bilateral amygdala, and parahippocampal gyrus), subcortical regions (bilateral caudate, claustrum, putamen, insula, and thalamus), occipital lobe (BA 7, 18, and 19), temporal lobe (BA 20 and 37), and the corpus callosum (CC). These regions were generally in coincidence with a volumetric studies by the voxel-based morphometry (VBM) method and revealed reduced density of gray and/or white matter in the corpus callosum, limbic system, frontal lobe, subcortical areas, temporal lobe, and the cerebellum [24–26]. However, these findings about lower integrity domains of current study were only somewhat similar with previous DTI studies, in which the frontal and temporal regions proved the general lower integrity domains with mTBI [27]. The studies utilizing voxel-based techniques showed the discrepant losses in the brain areas. In Lipton et al.'s work, the domains were CC, subcortical white matter, and internal capsules, bilaterally in chronic mTBI [28]. In another study, in the acute mTBI (≤2 weeks), the significant changes were mainly located in the frontal white matter, including the dorsolateral prefrontal cortex [29]. The FA attenuated regions were also found in the right temporal subcortical white matter including the inferior frontooccipital fasciculus in the subacute mTBI [30]. Obviously, the current findings included almost all regions of previous studies, potentially deriving from the more sensitive analysis method and assessment to the whole brain including white and gray matters. Notably, we observed the pronounced FA reduction in the thalamus, which was a key node in many of brain function networks [31] but was often overlooked in previous DTI studies. The damage to the thalamus-seeded structural connectivity is an important determinant of outcomes after TBI [32]. Our findings suggested a loss of integrity in the precuneus which served as an important node within the default mode network (DMN). The locations of thalamus and the precuneus provided us with a novel idea that perhaps the damage which resulted by TBI was not only the focal lesion but also the disconnection of brain network. Standing at this point, the following study will use the combination of multiple tractographical, analytical, and statistical methods to detect more tiny damage to the specific brain network in the whole brain range.

## 5. Conclusions

We demonstrated the sensitivity of DTI in identifying microstructural abnormalities in patients classified as “symptomatic” TBI with the minimal severity, no loss of consciousness, posttraumatic anterograde amnesia, and no contusions and microhemorrhage on conventional neuroimaging. A better understanding of the acute changes occurring following symptomatic TBI may increase our understanding of neuroplasticity and continuing degenerative changes, which. in turn, may facilitate advances in management and intervention. Future analyses will include additional examination of the relation of imaging changes to cognitive and functional outcome as well as multimodal imaging analyses of symptomatic TBI.

## Figures and Tables

**Table 1:** Demographic and injury characteristics of TBI.

Patient ID	Gender	Age	Education (years)	Days after injury	Causes
1	F	38	4	6	Assaults
2	M	36	15	1	RTA
3	M	19	13	1	Assaults
4	M	50	5	7	Assaults
5	M	21	7	5	Falls
6	F	29	15	7	RTA
7	M	48	1	7	Falls
8	M	42	6	7	RTA
9	M	46	9	4	RTA
10	M	26	12	7	Assaults
11	M	23	9	7	RTA
12	M	50	9	7	Assaults

ATBI: traumatic brain injury; M: male; F: female; RTA: road traffic accidents.

**Table 2:** Gray matter regions of significant FA reductions in 12 sTBI.

	Talairach			*t*	Voxels
	*x*	*y*	*z*	Value	
Caudate head					
L	−13	20	−1	2.40	128
Claustrum					
L	−31	3	7	2.88	31
R	36	−8	6	3.00	34
Insula					
L	−36	0	10	2.24	39
R	34	20	5	3.31	50
Putamen					
L	−25	−3	−2	3.24	138
R	23	−1	3	3.30	176
Thalamus					
L	−10	−8	15	2.99	297
R	15	−30	2	4.03	282
AN_Thalamus					
L	−10	−9	14	2.55	45
R	10	−6	13	2.79	61
MDN_Thalamus					
L	−7	−20	7	2.66	204
R	12	−19	6	3.65	184
Pulvinar_Thalamus					
L	−5	−23	9	2.92	166
R	17	−24	6	3.38	301
VLN_Thalamus					
L	−9	−8	6	1.96	67
R	11	−11	−17	2.47	66

sACC BA 25					
L	−7	17	−10	2.14	30
R	8	17	−10	2.20	47
pACC BA 32					
L	−12	36	17	3.67	71
R	10	37	19	2.96	80
Cigulate cortex BA 32/24					
L	−7	−7	39	2.23	218
R	9	−2	47	4.93	326
Amygdala					
L	−28	−6	−12	1.64	28
PH BA 36/37					
L	−23	−30	−12	3.84	185
R	23	−30	−4	3.14	143
PCC BA 23					
L	−1	−56	15	1.60	26
R	5	−47	24	3.37	199

OFC BA 10/47					
L	−10	−13	49	4.38	112
DLPFC BA 6/9					
L	−10	−13	49	4.38	112
R	5	−22	50	3.14	82

Lingual gyrus BA 18/19					
L	−17	−64	0	2.51	86
R	13	−52	3	3.45	149

Fusiform gyrus BA 37/20					
L	−44	−39	−17	2.36	45
R	32	−36	−17	1.27	24
R	20	−42	−7	1.39	21

AsTBI: symptomatic traumatic brain injury; LN: lentiform nucleus; AN: anterior nucleus; MDN: medial dorsal nucleus; VLN: ventral lateral nucleus; sACC: subgenual anterior cingulate cortex; pACC: perigenual anterior cingulate cortex; PH: parahippocampal gyrus; PCC: posterior cingulate cortex; OFC: orbitofrontal cortex; DLPFC: dorsolateral prefrontal cortex; BA: Brodmann area; PH: parahippocampus; AL: anterior lobe.

**Table 3:** White matter regions of significant FA reduction in 12 sTBI.

	Talairach			*t*	Voxels
	*x*	*y*	*z*	Value	
MFG					
L	−10	53	8	3.81	140
R	10	43	21	4.37	153
PG					
L	−46	−7	36	1.66	33
R	41	−10	38	2.55	52
SG					
L	−13	12	−11	3.45	62
R	9	15	−13	3.47	58
Subgyral					
L	−20	−7	56	3.05	282
R	11	−23	46	3.75	329

PH					
L	−30	−38	−5	4.96	537
R	32	−30	−11	4.12	461
PCC					
L	32	−58	15	2.11	33
R	32	−59	8	3.48	22
ACC					
L	−6	40	−4	3.43	145
R	13	44	−6	2.48	95
Cingulate cortex					
L	−12	−3	32	3.60	102
R	8	−1	46	3.71	173

Extranuclear					
L	32	−29	13	3.23	667
R	32	−14	15	3.90	487
Corpus callosum					
L	32	−14	18	2.05	23
R	32	−42	6	3.02	46
Insula					
L	32	−11	10	2.77	327
R	32	19	5	3.73	186

Cuneus					
L	32	−59	6	1.95	65
Lingual gyrus					
L	32	−58	3	3.09	283
R	32	−60	0	3.33	260

Precuneus					
L	−10	−57	35	4.88	29
R	12	−51	40	2.52	49

MTG					
L	32	−34	2	2.09	28

AMFG: middle frontal gyrus; PeCG: precentral gyrus; SG: subcallosal gyrus; PH: parahippocampal gyrus; PCC: posterior cingulate cortex; ACC: anterior cingulate cortex; LG: Lingual Gyrus; MTG: middle temporal gyrus.
